# Supplementary material for: Reporting delays of chikungunya cases during the 2017 outbreak in Lazio region, Italy
Source: PLoS Negl Trop Dis. 2023 Sep 14;17(9):e0011610. doi: 10.1371/journal.pntd.0011610 (PMC10501639; doi:10.1371/journal.pntd.0011610)
Supplement: S1 Text — (PDF) [file pntd.0011610.s001.pdf]

Supplementary materials

Reporting delays associated with the 2017 chikungunya outbreak in the Lazio region, Italy.

Mattia Manica[1, 2, 3], Giovanni Marini[2, 3], Angelo Solimini[4], Giorgio Guzzetta[1, 3], Piero Poletti[1, 3], Paola Scognamiglio[5], Chiara Virgillito[4], Alessandra della Torre[4], Stefano Merler[1, 3], Roberto Rosà[2, 6], Francesco Vairo[5], Beniamino Caputo[4]

[1]Center for Health Emergencies, Bruno Kessler Foundation, Trento, Italy
[2]Research and Innovation Centre, Fondazione Edmund Mach, San Michele all'Adige (TN), Italy.
[3]Epilab-JRU, FEM-FBK Joint Research Unit, Trento, Italy
[4]Department of Public Health and Infectious Diseases, Università di Roma SAPIENZA, Rome, Italy
[5]Regional Service for Surveillance and Control of Infectious Diseases (SERESMI)—Lazio Region, National Institute for Infectious Diseases "Lazzaro Spallanzani"; IRCCS, Rome, Italy
[6]Center Agriculture Food Environment, University of Trento, San Michele all'Adige (TN), Italy

Table of Contents

1. **_Outbreak identification and reporting system_** 2
2. **_Vector control interventions_** 3
3. **_Estimation of human infectious period from imported chikungunya cases in Italy_** 4
4. **_Sensitivity analysis on the estimation of human infectious period_** 4
5. **_References_** 5

1. Outbreak identification and reporting system

The Italian integrated national surveillance and response plan against *Aedes*-transmitted diseases such as chikungunya, dengue and Zika includes guidelines and protocols for the prevention, surveillance, control and reporting of imported and autochthonous cases as well as guidelines and protocols for entomological surveillance [1]. The national plan is compliant with EU regulation and adopts for each disease the current EU case definition.
The plan has been regularly updated over the years and many local public authorities have tailored and implemented it to specific local needs. Based on national and regional guidelines, reporting of suspected arbovirus cases to local Public Health authorities is mandatory. The reporting of a suspect case is usually made from a medical practitioner when treating a symptomatic individual that seek medical care.

The guidelines for reporting in effect at the time of the outbreak were the following [1]:

The medical practitioner that suspects an arboviral infection in subjects who meet the clinical and epidemiological case definitions reported below must:

- *i)* report it to the competent regional Public Health authority in charge of the epidemiological surveillance within 12 hours,
- *ii)* collect samples to send for laboratory confirmation according to a predefined diagnostic algorithm.

Samples must be sent either to the regional reference laboratories or to the national reference laboratory. If samples are sent to private laboratories, then the diagnosis has to be confirmed by a reference laboratory.
The medical practitioner carries out a preliminary recollection of case information when formulating the diagnosis of suspected case. The competent local Public Health authority in charge of the epidemiological surveillance is mandated to act when laboratory testing results in a confirmed or probable diagnoses.
In the event of positive laboratory tests (*ie* confirmed or probable case), the competent local Public Health authority must be immediately informed and report the findings to the Ministry of Health and the National Institute of Health within 12 hours.
Moreover, trained personnel must ensure that the following activities are implemented:

- *a)* carry out a more detailed epidemiological investigation collecting data on patients' age, sex, place of living, time of symptoms' onset, history of travel within Italy or abroad during the 15 days before symptoms' onset;
- *b)* inform the case, his/her relatives, and any individual living in the same household about the measure to adopt to reduce the risk of transmission;
- *c)* collect samples to send for laboratory confirmation;
- *d)* organize vector control measure that should be carried out within 24 hours after the notification of a probable or confirmed case;
- *e)* conduct a thorough environmental survey and epidemiological investigation to assess the presence of possible autochthonous cases and epidemic outbreaks (two or more cases occurring within a 30-day period in a restricted territorial area);
- *f)* report to the Ministry of Health and National Institute of Health.

Additionally, to contrast the spreading of the outbreak the local public health authority implemented a door-to-door active case finding in all households located inside a 200m radius from a probable or

confirmed case and active case finding in the community and in healthcare settings by general practitioners, paediatricians, in emergency rooms and hospitals admissions. Moreover, any subject being a relative, living in the same household or being a neighbor (living in radius of 200m from the case household) of a confirmed/probable case was notified as suspected case and tested. Finally, retrospective investigation to identify past infections was carried out and any subject reporting fever and joint pain during the previous 5 months was notified as suspected case and tested.

Case definition

Clinical criteria for suspected case:
Acute onset of fever and severe polyarthralgia (such as to limit normal activities daily activities), in the absence of other causes.

Epidemiological criteria for suspected case:
i) recent history of travel to an endemic country in the previous 15 days,
ii) being a relative, living in the same household or being a neighbor (living in radius of 200m from the case household) of a confirmed/probable case.

Laboratory criteria for probable case:
any suspected case that tested positive for anti- CHIKV IgM on a single serum sample.

Laboratory criteria for confirmed case:
any suspected case that tested positive for at least one of the following:
i) CHIKV PCR,
ii) anti- CHIKV IgM on a single serum sample confirmed by sero-neutralization,
iii) seroconverted from negative to positive or
iv) showed a fourfold increase of Ig titer in two subsequent samples taken at least 2 weeks apart.

2. Vector control interventions

Following national and regional guidelines, within 24 hours from the first notification a wide range of vector control measures were deployed by the competent authorities (ie the municipality in close collaboration with the regional public health service).

Vector control interventions included aerial spraying with pyrethroid insecticides, application on the vegetation of residual etofenprox-based insecticides and treating street drains with products based on mixed Bacillus thuringiensis var. israelensis and Bacillus sphaericus. Insecticide treatments were targeted at public areas (streets and parks) and private houses with gardens. Private houses were treated only when the owner allowed entering the premises. A private vector control company was hired specifically to perform reactive vector control interventions, *ie* larviciding and adulticiding.

Timeline of interventions
On September 7 vector control interventions aimed at knock down adult mosquito were carried out in a 200m buffer around the address of residence of the three notified case.
Despite new suspected cases being identified and/or confirmed, vector control activities were interrupted in the following days due to heavy precipitation events. Vector control intervention in public areas were resumed on September 12 where both adulticide and larvicide treatments were administered. In the same day also adulticide interventions in private areas (linked to epidemiological transmission) were resumed. Vector control interventions lasted up until the end of October [2].

3. Estimation of human infectious period from imported chikungunya cases in Italy

We estimated the human infectious period from imported chikungunya cases in Italy by assuming that the infectious period overlaps the symptomatic period and follows a truncated Poisson distribution. We used the function *fitdist*, distribution "ztpois", in the R package fitdistrplus ([3] version 1.1-11) resulting in a mean estimate of 5.27, standard deviation of 0.563.

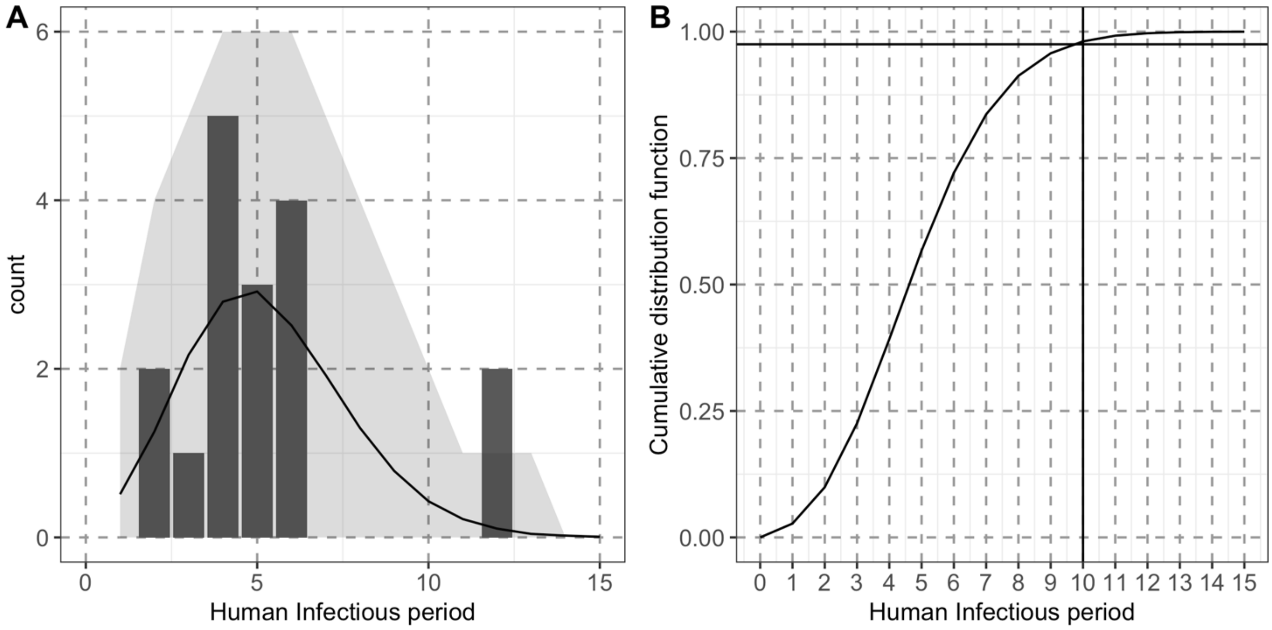

Fig A. *Left panel. Estimated frequency distribution of human infectious period. On the x-axis the human infectious period. On the y-axis the expected frequencies. Solid line represents the expected average frequency distribution of timeliness as estimated by the zero-truncated Poisson generalized linear model. The grey area represents the 95% confidence interval of the expected frequency. The solid bars represent the observed frequency distribution of human infectious period in the dataset. Right panel. Cumulative distribution function of the zero-truncated Poisson distribution. The horizontal line represents 0.95 probability level.*

4. Sensitivity analysis on the estimation of human infectious period

Using data records associated with imported cases [24], we estimated that the infectivity period lasted on average 5.3 days (95%CI: 4.2 – 6.4). Under the hypothesis that this overlaps the symptomatic period we assumed that the infectious period would last 5 days for 50% of symptomatic cases and would span from 1 to 10 days for 95% of symptomatic cases (Figure S1). We found that, on average 1.7% (95%CI: 0.5 – 3.2%) of cases had a notification time shorter than their infectious period BOD. This value increased to 32.3% (95%CI: 28.3 – 36.3%) AOD. Similarly, the number of days when onward transmission can occur avoided per person was 0.02 days (95%CI: 0 – 0.06) BOD and 0.75 (95%CI: 0.60 – 0.90) AOD, resulting on average 0.41 days (95%CI: 0.33 – 0.50) considering the whole period. This suggests an average percentage reduction in the number of days when onward transmission can occur of 0.4% (95%CI: 0 – 1.1%), 14% (95%CI: 11.6 – 16.5%) and 7.8% (95%CI: 6.3 – 9.3%), respectively.

5. References

[1] Ministry of Health. Italian National plan for the surveillance and control of Chikungunya, Dengue and Zika 2017. Ministry of Health; Accessed 10 July 2023. Available: http://www.salute.gov.it/portale/news/p3_2_1_1_1.jsp?lingua=italiano&menu=notizie&p=dalministero&id=3013

[2] European Centre for Disease Prevention and Control. Clusters of autochthonous chikungunya cases in Italy, first update – 9 October 2017. Stockholm: ECDC; 2017. Available: https://www.ecdc.europa.eu/sites/default/files/documents/RRA-chikungunya-Italy-update-9-Oct-2017.pdf

[3] Marie Laure Delignette-Muller, Christophe Dutang (2015) fitdistrplus: An R Package for Fitting Distributions. Journal of Statistical Software, 64(4), 1-34. DOI 10.18637/jss.v064.i04.

[4] Cauchemez S, Ledrans M, Poletto C, Quenel P, De Valk H, Colizza V, et al. Local and regional spread of chikungunya fever in the Americas. Eurosurveillance. 2014;19. doi:10.2807/1560-7917.ES2014.19.28.20854
