# Supplementary material for: Reporting delays of chikungunya cases during the 2017 outbreak in Lazio region, Italy
Source: PLoS Negl Trop Dis. 2023 Sep 14;17(9):e0011610. doi: 10.1371/journal.pntd.0011610 (PMC10501639; doi:10.1371/journal.pntd.0011610)
Supplement: Attachment — Submitted filename: Response_reviewers.docx [file pntd.0011610.s002.docx]

[1] A letter containing a detailed list of your responses to the review comments and a description of the changes you have made in the manuscript. Please note while forming your response, if your article is accepted, you may have the opportunity to make the peer review history publicly available. The record will include editor decision letters (with reviews) and your responses to reviewer comments. If eligible, we will contact you to opt in or out.

[2] Two versions of the revised manuscript: one with either highlights or tracked changes denoting where the text has been changed; the other a clean version (uploaded as the manuscript file).

***********************


Reviewer's Responses to Questions

Reviewer #1: The methods are appropriate for the analyses conducted. Specific clarifications are noted:

**Response:** We thank the reviewer for their appreciation of our work. We have updated the manuscript integrating all of their suggestions.

1.      To help understand the presented information on timing of infection reporting relative to outbreak detection it would be useful for readers to understand some of the details of the reporting system or systems used in different regions of Italy. Is chikungunya infection reporting mandatory? Please describe the SERESMI reporting system so persons outside Italy can better appreciate the context. Who reports the case information and what are the details of the information which are requested?

**Response:** We apologize for the lack of details. We have now expanded the manuscript and the supplementary materials (section 1 “Outbreak identification and reporting system”) to include a more detailed account of the reporting system at both national and regional level. We also included new references that detail the surveillance and response system in the Lazio region.

Main text

Line 128 [in the highlighted version] “The surveillance system in place in the Lazio region in 2017 included different surveillance tools: passive surveillance relying on medical practitioners reporting suspected cases, laboratory-based surveillance, and syndromic surveillance [20]. Based on national and regional guidelines, reporting of suspected arbovirus cases to local Public Health authorities is mandatory [14,17,21].”

Line 144 “The medical practitioner that suspects an arboviral infection in subjects who meet the clinical and epidemiological case definitions reported below must report it to the competent regional Public Health authority in charge of the epidemiological surveillance within 12 hours and collect samples to send for laboratory confirmation. “

Line 153 “In the event of positive laboratory tests, the case must be immediately reported by the health authority to the administrative body of the Region and within 12 hours to the Ministry of Health and the National Institute of Health. The guidelines for reporting in effect at the time of the outbreak are included in supplementary.”

The information requested are available in greater detail in the now cited literature:

- European Centre for Disease Prevention and Control. Clusters of autochthonous chikungunyacases in Italy, 14 September 2017. Stockholm: ECDC; 2017. Available: <https://www.ecdc.europa.eu/sites/default/files/documents/14-Sep-2017-RRA-Chikungunya-Italy-revised.pdf>
- European Centre for Disease Prevention and Control. Clusters of autochthonous chikungunya cases in Italy, first update – 9 October 2017. Stockholm: ECDC; 2017. Available: <https://www.ecdc.europa.eu/sites/default/files/documents/RRA-chikungunya-Italy-update-9-Oct-2017.pdf>
- Ministry of Health. Italian National plan for the surveillance and control of Chikungunya, Dengue and Zika 2017. [only Italian version available]. Available from: <http://www.salute.gov.it/portale/news/p3_2_1_1_1.jsp?lingua=italiano&menu=notizie&p=dalministero&id=3013>
- Venturi G, Di Luca M, Fortuna C, Remoli ME, Riccardo F, Severini F, et al. Detection of a chikungunya outbreak in Central Italy, August to September 2017. Eurosurveillance. 2017;22. doi:10.2807/1560-7917.ES.2017.22.39.17-0064 (reference 16 in the revised version of the manuscript)
- Vairo F, Mammone A, Lanini S, Nicastri E, Castilletti C, Carletti F, et al. Local transmission of chikungunya in Rome and the Lazio region, Italy. Roques P, editor. PLOS ONE. 2018;13: e0208896. doi:10.1371/journal.pone.0208896 (reference 14 in the revised version of the manuscript)
- Vairo F, Di Pietrantonj C, Pasqualini C, Mammone A, Lanini S, Nicastri E, et al. The Surveillance of Chikungunya Virus in a Temperate Climate: Challenges and Possible Solutions from the Experience of Lazio Region, Italy. Viruses. 2018;10: 501. doi:10.3390/v10090501

2.      On page 11, the authors state that notification of infection can lead to a reduction in infectious days. Could the authors clarify what is meant here? Is this the cumulative infectious because of the presumed impact of case recognition on the overall number of cases infected? For each infected individual there would be no change/reduction in the infectious days, but at the population level there could be.

**Response:** We apologize for the misleading wording used here. We agree with the reviewer that the number of infectious days experienced by each infected individual does not depend on its notification. Notification can reduce the probability of onward transmission by triggering the enactment of vector control interventions and personal protection measures. Under the assumption that after notification the probability of onward transmission is zero due to the implementation of optimal vector control and/or protective measures, then the individual reduction in the probability that onward transmission can occur is equivalent to a reduction of infectious days at the population level. To help readers’ comprehension we have now reworded the sentence “reduction in infectious days” as follows: “reduction in the probability that onward transmission can occur” or “reduction in the number of infectious days at population level”.

3.      The assumption that the symptomatic period and infectious period overlap may not be correct. In most situations the infectious period would precede the infectious period and then seroconversion and reduced infectiousness would be evident during the most symptomatic phase of infection.

**Response:** We thank the reviewer for raising this point. We agree with the reviewer’s comment that our assumption (infectiousness overlapping the symptomatic period) may not be correct. We moved our previous analysis in supplementary as a sensitivity analysis and included a new analysis informed by data obtained from Cauchemez et al, 2014 (quote: “On the basis of viremia time profile, a human host was assumed to be infectious for 5 to 8 days, with infectivity starting 1 to 2 days before symptoms.”). Therefore, for each case we assigned the start of the infectious period with respect to the date of symptom onset by sampling one or two days before (with equal probability 0.5) and then we sampled the length of the infectious period uniformly between 5 to 8 days. We then compared the estimated date of end of the infectious period with the observed date of notification. Results of this analysis showed a slightly lower percentage of cases notified before the end of their infectious period (17% compared to the previously estimated 18.3%) and subsequently lower percentage reduction of infectious days at population level (4.4% compared to the previously estimated 7.8%). Finally, we included the following sentence as a limitation of our approach:

Line 333 “In addition, the lack of information on the individual dates of infection and duration of infectiousness may have also led to an overestimation of the reduction in the number of infectious days at population level.”

- Cauchemez S, Ledrans M, Poletto C, Quenel P, de Valk H, Colizza V, Boëlle P Y. Local and regional spread of chikungunya fever in the Americas. Euro Surveill. 2014;19(28):pii=20854. https://doi.org/10.2807/1560-7917.ES2014.19.28.20854

Reviewer #2: The study has a clearly articulated hypothesis with subsequent analysis that adequately tests that hypothesis. The population studies is clearly described, and the samples used in analysis are aa comprehensive ans inclusive as possible given reporting limitations. The paper in its current form sufficiently answers the research question.

**Response:** We thank the reviewer for his/her kind assessment of our work.

Reviewer #3: 1. Reporting delay can be the result of patients delaying their presenting for care, especially when the symptoms are mild, or delay in sending the specimens for testing or delay in the laboratory sending the results back to the patient and the health provider.  It is also not known how the results gets reported to the surveillance system.  It would be useful if the authors can briefly describe how the outbreak emerged, how the cases are detected (from the surveillance system or from clinicians in the community or both) and what is involved in cases processing from suspect cases to probable and confirmed cases (was testing done at surveillance sites or were specimens transported to laboratories?) and how the cases are reported to the surveillance system?

**Response:** We apologize for the lack of clarity on this point. We have now expanded the introduction, included in supplementary materials more details on the emergence of the outbreak as well as the flow of information in the reporting system (section 1 “Outbreak identification and reporting system”) and added relevant references.

Line 117 “The outbreak in the Lazio region was identified when the National Reference Laboratory for Arboviral Infections at the National Institute of Public Health and the National Institute for Infectious Diseases analyzed serum and urine samples from a cluster of three suspected cases [16]. All cases resulted IgM positive on 6 and 7 September 2017 and were confirmed through a neutralisation test [14,16,17]. All cases showed symptoms (i.e. high fever above 38° C, severe joint pain and skin rash) but only one case was still symptomatic at the time of laboratory testing. None of the cases had an history of travel in endemic area in the past 15 days, they all lived in the same household and the onset of symptoms happened during their holiday in the coastal area of Anzio [14,16,17].”

Line 128 “The surveillance system in place in the Lazio region in 2017 included different surveillance tools: passive surveillance relying on medical practitioners reporting suspected cases, laboratory-based surveillance, and syndromic surveillance [20]. Based on national and regional guidelines, reporting of suspected arbovirus cases to local Public Health authorities is mandatory.”

Line 144 “The medical practitioner that suspects an arboviral infection in subjects who meet the clinical and epidemiological case definitions reported below must report it to the competent regional Public Health authority in charge of the epidemiological surveillance within 12 hours and collect samples to send for laboratory confirmation. “

Line 153 “In the event of positive laboratory tests, the case must be immediately reported by the health authority to the administrative body of the Region and within 12 hours to the Ministry of Health and the National Institute of Health. The guidelines for reporting in effect at the time of the outbreak are included in supplementary.”

The information requested are available in greater details in the now cited literature:

- European Centre for Disease Prevention and Control. Clusters of autochthonous chikungunyacases in Italy, 14 September 2017. Stockholm: ECDC; 2017. Available: <https://www.ecdc.europa.eu/sites/default/files/documents/14-Sep-2017-RRA-Chikungunya-Italy-revised.pdf>
- European Centre for Disease Prevention and Control. Clusters of autochthonous chikungunya cases in Italy, first update – 9 October 2017. Stockholm: ECDC; 2017. Available: <https://www.ecdc.europa.eu/sites/default/files/documents/RRA-chikungunya-Italy-update-9-Oct-2017.pdf>
- Ministry of Health. Italian National plan for the surveillance and control of Chikungunya, Dengue and Zika 2017. [only Italian version available]. Available from: <http://www.salute.gov.it/portale/news/p3_2_1_1_1.jsp?lingua=italiano&menu=notizie&p=dalministero&id=3013>
- Venturi G, Di Luca M, Fortuna C, Remoli ME, Riccardo F, Severini F, et al. Detection of a chikungunya outbreak in Central Italy, August to September 2017. Eurosurveillance. 2017;22. doi:10.2807/1560-7917.ES.2017.22.39.17-0064 (reference 16 in the revised version of the manuscript)
- Vairo F, Mammone A, Lanini S, Nicastri E, Castilletti C, Carletti F, et al. Local transmission of chikungunya in Rome and the Lazio region, Italy. Roques P, editor. PLOS ONE. 2018;13: e0208896. doi:10.1371/journal.pone.0208896 (reference 14 in the revised version of the manuscript)
- Vairo F, Di Pietrantonj C, Pasqualini C, Mammone A, Lanini S, Nicastri E, et al. The Surveillance of Chikungunya Virus in a Temperate Climate: Challenges and Possible Solutions from the Experience of Lazio Region, Italy. Viruses. 2018;10: 501. doi:10.3390/v10090501

2.      What were the criteria for establishing Sept 6 2017 as the “date of first notification”? was it based on a cluster of confirmed cases?  What measures were put into place once an outbreak is officially declared?  Were family members of cases tested for additional case finding?         

**Response:** The date of first notification is defined as the date when the first suspected chikungunya case was reported to the competent Public Health authorities (i.e., the Lazio regional public health service (SERESMI)) which in turn must implement within 24 hours all the following mandatory actions: laboratory confirmation of diagnosis, enactment of reactive vector control measures, and notification to the Ministry of Health of the detection of an arbovirus case. In 2017, outbreak identification (on September 6) was triggered by a cluster of three individuals without a history of travel in endemic areas that showed symptoms consistent with an arbovirus infection. In response to the detection of chikungunya cases (resulting positive to IgM analysis on serum and urine samples and then confirmed by neutralisation test), reactive measures were immediately put into place. The surveillance system was strengthened through the dissemination of a dedicated case definition and contact-tracing activity were started. In Anzio, a door-to-door active case finding was implemented in all households located inside a 200m radius from a probable or confirmed case. Any subject being a relative, living in the same household or being a neighbor (living in radius of 200m from the case household) of a confirmed/probable case was notified as suspected case and tested. Additionally, any subject reporting fever and joint pain during the previous 5 months was notified as a suspected case and tested. The epidemiological investigation collected data on patients’ age, sex, place of living, time of symptoms’ onset, history of travel within Italy or abroad during the 15 days before symptoms’ onset.

We have now included the following statements in the main text that summarized such information and provide a full account of the public health response in the supplementary materials (section 1 Outbreak identification and reporting system and section 2 Vector control interventions).

Main text

Line 117: “The outbreak in the Lazio region was identified when the National Reference Laboratory for Arboviral Infections at the National Institute of Public Health and the National Institute for Infectious Diseases analyzed serum and urine samples from cluster of three suspected cases [16]. All cases resulted IgM positive on 6 and 7 September 2017 and were confirmed confirmation through a neutralisation test [14,16,17]. All cases showed symptoms (ie high fever above 38° C, severe joint pain and skin rash) but only one case was still symptomatic at the time of laboratory testing. None of the cases had an history of travel in endemic area in the past 15 days, they all lived in the same household and the onset of symptoms happened during their holiday in the coastal area of Anzio [14,16,17]. After the identification of the outbreaks, health authorities implemented blood safety restrictions (e.g.: suspension of blood collection) until 1 December 2017 [15,18]. and reactive vector control measures by aerial spraying with pyrethroid insecticides, residual etofenprox-based insecticides and larvicide in street drains in both public areas and private houses [16,19].”

3.      Vector Control:  Since there is no treatment or vaccines for Chikungunya, prevention and control interventions generally rely on case-based interventions (mosquito spraying and other vector control measures around the residence of cases)., the proportion of asymptomatic but infectious individuals in a community would have a varying impact on the effectiveness of the outbreak response.  Were community-wide spraying of public spaces part of the vector control in addition to interventions around the place of residence of notified cases?

**Response:** Following national and regional guidelines, within 24 hours from the first notification a wide range of vector control measures were deployed by the competent authorities (i.e., the municipality, in close collaboration with the regional public health service).

Vector control interventions included aerial spraying with pyrethroid insecticides, application on the vegetation of residual etofenprox-based insecticides and treating street drains with products based on mixed Bacillus thuringiensis var. israelensis and Bacillus sphaericus. Insecticide treatments were targeted at public areas (streets and parks) and private houses with gardens. Private houses were treated only when the owner allowed entering the premises. On September 7 vector control interventions were carried out in a 200m buffer around the address of residence of every case, interrupted due to heavy rainfall and then resumed on September 12 to include public and private areas. A private vector control company was hired specifically to perform reactive vector control interventions, i.e., larviciding and adulticiding. Two teams of four operators carried out mandatory vector control from the 7 September to the end of October as well as area-wide larvicide application and insecticide sprayings.

We have now included the following sentences in the main text on line 125 ”After the identification of the outbreaks, health authorities implemented blood safety restrictions (e.g.: suspension of blood collection) until 1 December 2017 [15,18]. and reactive vector control measures by aerial spraying with pyrethroid insecticides, residual etofenprox-based insecticides and larvicide in street drains in both public areas and private houses [16,19].”

We also included a detailed section regarding vector control interventions in the supplementary and added the relevant reference in the main text.

- European Centre for Disease Prevention and Control. Clusters of autochthonous chikungunyacases in Italy, 14 September 2017. Stockholm: ECDC; 2017. Available: <https://www.ecdc.europa.eu/sites/default/files/documents/14-Sep-2017-RRA-Chikungunya-Italy-revised.pdf>
- European Centre for Disease Prevention and Control. Clusters of autochthonous chikungunya cases in Italy, first update – 9 October 2017. Stockholm: ECDC; 2017. Available: <https://www.ecdc.europa.eu/sites/default/files/documents/RRA-chikungunya-Italy-update-9-Oct-2017.pdf>

Reviewer #1: The results align with the planned analysis and no deficiencies are noted. Figures are appropriate. Supplemental figures are not included and could not be reviewed.

**Response:** We do apologize for the inconvenience. Supplementary figures are now included in the revised version of the manuscript.

Reviewer #2: Results are completely presented and figures clear and aesthetically pleasing.

Reviewer #3: The results are clearly presented and the figures are of sufficient quality for clarity.

**Response:** We thank the reviewers for their positive comments.

Reviewer #1: The conclusions are appropriate and supported by the data. The most relevant limitations are clearly stated.

The primary deficiency is that specific recommendations on how to improve the process or timeliness of infection reporting in Italy are not provided. This additional specificity would improve the practical relevance of the analysis and findings.

**Response:** We thank the reviewer for the comment. We have now added a short paragraph in the discussion that includes recommendations to improve the timeliness of infection reporting.

Line 346 “Early detection of imported chikungunya cases as well as the initial autochthonous transmission events is of the utmost importance to contain or even prevent the spread and size of any future chikungunya outbreak [23,31]. The aim of improving detection and notification timeliness can be achieved only by a sustained and coordinated effort involving many activities. For instance, it is essential not only to raise awareness on chikungunya infection in the general population and in individuals travelling to and from endemic countries but also provide information and training on diagnosis and reporting for medical practitioners. Moreover, medical practitioners should be supported by developing improved diagnostic algorithms as well as improving the information flow by minimizing the time delay between any response steps in the public health surveillance process. An important surveillance tool could be provided by syndromic surveillance where clearly defined sets of symptoms could be used to flag suspected transmission events. Also, novel data sources such as search generated data could be potentially integrated in the surveillance system in order to enhance the early detection of viral transmission. Another critical aspect in reducing reporting delay involves speeding up laboratory procedure both in national reference laboratories as well as in first-line responding hospital laboratories. This can be achieved by strengthening laboratory capability to face increased workload due to increased testing and by increasing laboratory detection capacity challenged by the paucity of available commercial kits, the cross-reactivity between related arboviruses affecting serology testing and the short viremia period. Finally, routine evaluation of reporting performances should be planned and routinely carried out to ensure the surveillance system preparedness and responsiveness.”

Reviewer #2: The conclusions are supported by the results and the authors provide helpful context that help the reader appreciate the public health significance of the findings.

**Response:** We thank the reviewer for the positive comment.

Reviewer #3: The discussion is good but it is not clear how the authors presented the quantitation of the health burden. what is the definition of health burden and how was that quantitated?
What are the limitations of their study - did they provide readers with a better understanding of reporting delays and how that impacted the final health burden of this outbreak compared to previous studies?

**Response:** We apologize for the unclear definition of health burden used in the abstract. We have now reworded as follows: “Here, we provide estimates of reporting delays during an emerging arboviral outbreak and indications on how delays may have impacted onward transmission”.

We hope that our analysis shed a light on an important but often overlooked aspect of arbovirus surveillance, i.e., the delay between infection and notification of cases. Previous modelling work on the same outbreak has shown that halving the time delay between symptom onset and intervention would have had a smaller impact (in terms of reduction of cases) than anticipating the detection of the outbreak by one or two generations of infection (12% against 44% and 71%, respectively; Guzzetta et al, 2020). Similarly, a recent analysis of the French chikungunya outbreaks suggested that a 10-days earlier intervention would have reduced the final outbreak size by 30–40% fewer cases. We have included this information in discussion (line 323).

However, case detection remains challenging in Europe due to the low prevalence and sporadic occurrence of Aedes-transmitted arbovirus infections.

- Guzzetta G, Vairo F, Mammone A, Lanini S, Poletti P, Manica M, et al. Spatial modes for transmission of chikungunya virus during a large chikungunya outbreak in Italy: a modeling analysis. BMC Med. 2020;18: 226. doi:10.1186/s12916-020-01674-y
- Jourdain F, de Valk H, Noël H, Paty M-C, L’Ambert G, Franke F, et al. Estimating chikungunya virus transmission parameters and vector control effectiveness highlights key factors to mitigate arboviral disease outbreaks. Rodo X, editor. PLoS Negl Trop Dis. 2022;16: e0010244. doi:10.1371/journal.pntd.0010244

Line 275:  What is meant by,” …..and the short radius of treatment compared to the potential distribution of transmission distances”?  treatment? Please explain.

**Response:** We apologize for the lack of clarity. Here, treatment is used as an abbreviation of vector control treatment. A previous study analyzing the transmission chain in the Anzio outbreak (Guzzetta et al 2020) showed that a non-negligible share of infections (i.e., 45.1%) are transmitted wider apart (>1 km) than the distance within which reactive vector control took place, suggesting that reactive vector control based on current guidelines (200m buffer) might still allow the seeding of a significant number of secondary clusters in untreated areas. We have now rephrased the sentence as follow: “Underlying reasons were the limited effectiveness of control interventions and the potentially wider distribution of transmission distances compared to the area targeted by reactive vector control interventions [Guzzetta et al 2020]”.

- Guzzetta G, Vairo F, Mammone A, Lanini S, Poletti P, Manica M, Rosa R, Caputo B, Solimini A, Torre AD, Scognamiglio P, Zumla A, Ippolito G, Merler S. Spatial modes for transmission of chikungunya virus during a large chikungunya outbreak in Italy: a modeling analysis. BMC Med. 2020 Aug 7;18(1):226. doi: 10.1186/s12916-020-01674-y

Reviewer #1: Page 5, Line 147 – dichotomous rather than dichomotic
Page 6, Line 161 – truncated rather than truncate
Page 11, Line 236 – ‘the symptomatic period’ rather than symptom

Reviewer #2: Line 76 - chikungunya should be decapitalized for consistency internally and with the existing literature
Line 153 - analysis should read analyses
Line 180 - figure caption shoukd state “events” rather than “evens”

**Response:** We revised accordingly.

Reviewer #1: The supplemental materials do not appear available to review and so were not reviewed.

**Response:** We do apologize for the inconvenience. Supplementary figures are now included in the revised version of the manuscript.

Reviewer #2: This study answers an important epidemiological question using rigorous methods. The results are presented in a clear manner, elucidating important findings without overstating. The paper provides helpful insight that can help the scientific community better understand the ramifications of public health campagins during disease outbreaks.

**Response:** We thank the reviewers for the positive comment.

Reviewer #3: This manuscript describes a study to quantify reporting delays of cases and assess the impact of these delays on the final health burden during an outbreak of Chikungunya in Italy.  Reporting delays of infectious disease outbreaks have major impact on the development and implementation of disease prevention and control strategies.  Hence this type of study should be a high priority during inter-epidemic periods to inform how surveillance and health systems should be optimised for managing future outbreaks.  The aims are clearly stated and  the methods and results are well described.  However, there is insufficient information on aspects of the outbreak and the control strategies for readersts to interpret the results and draw conclusions.

**Response:** We thank the reviewers for all comments that helped improving the manuscript. We hope that the revised version of the main text and associated supplementary material now report all the information needed to help readers interpret results and draw conclusions.
